# Differences in menstrual symptoms and practices among university students: A comparison of Turkish Republics, Middle Eastern, and African Countries

**DOI:** 10.12669/pjms.41.8.12042

**Published:** 2025-08

**Authors:** Duygu Yeşilfidan, Serkan Köksoy, Filiz Adana, Safiye Özvurmaz, Ceren Varer Akpinar, Aliye Mandiracioglu

**Affiliations:** 1 Duygu Yeşilfidan, Department of Public Health Nursing, Faculty of Nursing, Aydın Adnan Menderes University, Aydın, Türkiye; 2 Serkan Köksoy, Department of Public Health Nursing, Faculty of Health Sciences, Burdur Mehmet Akif Ersoy University, Burdur, Türkiye; 3 Filiz Adana, Department of Public Health Nursing, Faculty of Nursing, Aydın Adnan Menderes University, Aydın, Türkiye; 4 Safiye Özvurmaz, Department of Public Health Nursing, Faculty of Nursing, Aydın Adnan Menderes University, Aydın, Türkiye; 5 Ceren Varer Akpinar, Department of Public Health, Faculty of Medicine, Giresun University, Giresun, Türkiye; 6 Aliye Mandiracioglu, Department of Public Health, Faculty of Medicine, Ege University, İzmir, Türkiye

**Keywords:** Culture, International Students, Menstruation, Menstrual hygiene, Reproductive health

## Abstract

**Objective:**

This study aimed to determine the differences in menstrual symptoms and practices among university students from Turkish Republics, African, and Middle Eastern countries.

**Methodology:**

This study is a cross-sectional research conducted among international and domestic female students studying at a university in western Turkey. The sample consisted of 714 female students determined by the sampling method for a known population. The data of the study were collected online in the 2022-2023 academic year via the Personal Information Form and Menstrual Symptom Questionnaire. Mann-Whitney U test was used for the comparison of two independent groups, the Kruskal Wallis H test was used for the comparison of more than two independent groups, and the chi-square test was used for the comparison of categorical data.

**Results:**

The mean age of the students participating in the study was 21.49±1.80 years and 81.1% of the students were from Turkish Republics, 14.6% were Middle Eastern, and 4.3% were African. 98.7% of the students were single, and 90.1% had lived in urban areas. Turkish Republic students had less frequency of product changing during menstruation, more information about perineal hygiene during menstruation, use of cotton underwear, proper cleansing in the toilet, and standing showering during menstruation compared to other students(p<0.05). African and Middle Eastern students reported a higher frequency of not mentioning the continuation of sexual intercourse during menstruation compared to Turkish Republic students(p<0.05).

**Conclusions:**

The research has shown that there are international differences in the symptoms and practices related to menstruation among female students from different cultures and nationalities.

## INTRODUCTION

Menstruation plays a significant role as a function of female reproduction and is closely associated with women’s health.^1^ Although menstruation is a universal biological process, cultural beliefs significantly shape women’s perceptions and practices. In some societies, menstruating women are viewed as unclean and restricted from activities like bathing, sports, or medical procedures. Misconceptions—such as frequent pad changes causing excessive bleeding—may disrupt daily life and increase stress, potentially affecting menstrual health.^1^-^3^ Van Eijk et al. reported various challenges in maintaining menstrual hygiene among women due to the lack of water, privacy, soap, buckets, and sanitary/plumbing facilities.^4^ The same study indicated that in many developing countries, there is a significant lack of knowledge about menstrual hygiene among women, and many hold misconceptions about menstruation.

Women in low and middle-income countries often struggle to access information about menstruation and fail to learn about hygiene practices during their menstrual period. Additionally, the cost of products presents another challenge. This situation can lead them experiencing more symptoms and issues related to menstruation.^4^-^6^

Generally, international students often face numerous challenges (such as unfamiliar food and living conditions, financial difficulties, and barriers related to language and culture) while pursuing higher education in a new environment. Particularly for international female students, understanding the factors that could change menstruation symptoms during the acculturation period can help alleviate the negative impacts of their experiences and enhance their productivity in the host environment.^2^,^7^

In Turkey, foreign students are enrolled at various levels of education. Especially, international programs at universities allow many students from different cultures to study together. As of 2022, there are over 300,000 international students from 198 countries studying at universities in Turkey.^8^ University students represent a young, dynamic, skilled, motivated, and innovation-friendly population, making studies in this group pioneering societal change and development. Therefore, the period of university education offers significant opportunities to identify and develop health-related practices for young women in education. In Turkey, the majority of students are from the Turkic Republics, Eastern Europe, the Middle East, and Africa. It is crucial to identify menstruation symptoms and practices among young women from these countries, which are mostly low-income and have unique cultural characteristics. However, the literature lacks studies describing the prevalence of in menstruation symptoms among international female students studying in Turkey. The purpose of this research was to identify the differences in menstruation-related symptoms and practices among international university students.

## METHODOLOGY

This study is a cross-sectional research conducted among international and domestic female students studying at a university in western Turkey. The population of the study consists of 1,100 international and 12.064 domestic female students. The sample size was calculated as 632 using α=0.05 and 99% power, and it was increased by 15% to 727 students to account for potential data losses. However, due to incomplete data from 13 students, the study was completed with 714 participants (579 from Turkic Republics, 104 from the Middle East, and 31 from Africa).

Data were collected online via Google Forms during the 2022–2023 academic year. The survey link was distributed through student communication platforms (e.g., WhatsApp). Participants could access the questionnaire only after providing informed consent.

### Ethical consideration:

Prior to the research, ethical approval (Approval No: 2022/941, Date: December 7, 2022) and informed consent from the participants were obtained. The research was conducted in accordance with the Declaration of Helsinki.

### Inclusion criteria:

- For the study were the ability to understand and write in Turkish, being aged 18 and over

### Exclusion criteria:

- The absence of menstruation due to any health problem.
- Those who incompletely filled out the data collection forms were excluded from the study.
- The countries of origin were divided into three groups (Turkic Republics, Middle Eastern, and African) and assessed, and Turkish students were also included in the Turkic Republics group.

### Data collection tools:

The data collection tools consist of a personal information form and the Menstrual Symptom Questionnaire (MSQ).

### Personal information Form:

This Form includes a total of 22 questions designed to determine the students’ sociodemographic characteristics, menstrual histories, and practices related to menstruation according to their cultures.^1^-^3^,^7^

### Menstrual Symptom Questionnaire

### (MSQ):

The Menstrual Symptom Questionnaire (MSQ), developed by Chesney and Tasto assesses the severity of menstrual symptoms. Its Turkish adaptation was validated by Güvenç et al..^9^ The 22-item Likert-type scale includes three subdimensions: Negative Effects/Somatic Complaints (items 1–13), Menstrual Pain (14–19), and Coping Methods (20–22), with scores ranging from 22 to 110. Cronbach’s alpha was 0.86 in the original study and 0.87 in the current research (subdimensions: 0.81, 0.80, 0.59, respectively).

### Statistical analysis:

The research data were analyzed using SPSS 25.0 at a 95% confidence interval and a significance level of 0.05. The normal distribution of the data was tested using the Kolmogorov-Smirnov analysis. The Mann-Whitney U test was used for comparing two independent groups, while the Kruskal-Wallis H test was used for comparing more than two independent groups. The Chi-Square test (Pearson Chi-Square, Likelihood Ratio) was utilized for comparing categorical data. Bonferroni correction and Tamhane’s test were performed to determine differences between groups in the Chi-Square and Kruskal Wallis H tests.

## RESULTS

The students participating in the study were found to be from 15 different nationalities, with the majority being from Turkey (51.8%), Turkmenistan (12.0%), and Syria (6.6%). It was found that 81.1% of them were from Turkic Republics, 98.7% were unmarried, and 90.1% had lived in urban centers. The average age of the students was 21.49±1.80 years.

Table-I presents the average scores for the Menstrual Symptom Questionnaire and Sub-dimensions. According to the analysis, the total MSQ score of students from the Turkic Republics was found to be higher compared to students from the Middle East (8.130;0.017); also, the average scores for the Menstrual Pain Sub-dimension were higher than those of other students (30.740;0.000).

**Table-I:** Menstrual Symptom Questionnaire and Sub-dimensions average scores among university students from Turkic Republics, African, and Middle Eastern Countries

Menstrual Symptom Questionnaire and Sub-dimensions Scores	Turkic Republics (n=579)			Middle East (n=104)			African (n=31)			Statistics; p
	*χ̅*±*S*	Median	1st Quarter; 3rd Quarter	*χ̅*±*S*	Median	1st Quarter; 3rd Quarter	*χ̅*±*S*	Median	1st Quarter; 3rd Quarter	
Negative Effects/Somatic Complaints	39.81±8.63	40.00	35.00;45.00	39.06±6.47	39.00	35.25; 42.00	38.35±4.77	37.00	36.00; 42.00	2.037;0.361
Menstrual Pain	20.37±5.12	20.00	17.00; 24.00	17.82±3.08	17.50	16.00; 19.00	18.35±3.34	18.00	16.00; 21.00	30.740;0.000
Methods of Coping with Menstrual Pain	8.51±2.81	8.00	7.00; 10.00	8.53±2.09	8.00	7.00; 10.00	8.58±1.92	8.00	7.00; 10.00	0.097;0. 953
MSQ Total	68.70±14.11	68.00	61.00-77.00	65.43±10.34	65.00	59.00; 70.00	65.29±8.52	63.00	61.00; 70.00	8.130;0.017

n: Number of participants; *χ̅*: Arithmetic mean; SD: Standard deviation; p: significance level. In this table, the Kruskal Wallis-H and Tamhane tests were used.

About 54.2% of students from the Turkic Republics reported that the onset of menstruation in girls is considered a normal and discussable situation in their families, while 26.9% stated that it is a condition that should be kept secret within their families. Another 3.5% of the students reported that female genital mutilation (FGM) is practiced in their countries, and 2.2% reported having undergone FGM themselves. These findings, respectively, for Middle Eastern students were 7.7%; 61.95%; 2.9%, and 2.9%, while for African students the figures were 29%; 54.8%, and 3.2% (Table-II).

**Table-II:** Comparison of reproductive health characteristics from a cultural perspective among university students from Turkic Republics, African, and Middle Eastern Countries.

Reproductive Health Characteristics From A Cultural Perspective / Regions		Turkic Republics		Middle Eastern		African		Statistics; p
		n	%	n	%	n	%	
What does the onset of menstruation mean for girls in your family?	It is a normal and discussable situation	314	54.2	8	7.7	9	29.0	No comparison has been made
	It is a situation that must be secret	156	26.9	64	61.5	17	54.8	
	It is a blissful state	47	8.1	5	4.8	-	-	
	It is shameful	42	7.3	22	21.2	3	9.7	
	It is a burden	2	0.3	-	-	1	3.2	
	It is a curse	1	0.2	-	-	-	-	
	Unanswered	17	2.9	5	4.8	1	3.2	
	Total	579	100	104	100	31	100	
Is female genital mutilation practiced in your country?	Yes	20	3.5	3	2.9	1	3.2	0.093;0.954 (LH)
	No	559	96.5	101	97.1	30	96.8	
	Total	579	100	104	100	31	100	
Have you been subjected to female genital mutilation?	Yes	13	2.2	3	2.9	-	-	1.586;0.453 (LH)
	No	566	97.8	97.1	30	31	100	
	Total	579	100	104	100	31	100	

n: Number of participants; %: percentile; *χ̅*: Arithmetic mean; SD: Standard deviation; p: significance level; LH: Likelihood Ratio. In this table, χ^2^: Chi-square test has been used.

No significant difference was found in the comparison of the prevalence of female genital mutilation practiced in the countries of the students participating in the study and the instances of female genital mutilation performed on the students themselves, according to their countries (Table-II).

The menstrual experiences of students according to regional divisions are observed in Table-III. Students from Turkic Republics reported that 35.1% felt nothing when they first menstruated. Those who received information about menstruation before it began constitute 84.8% and 82% stated they received information about perineal hygiene during menstruation, and 74.4% reported that this information was obtained from their mothers. 82.2% of the students experience regular menstruation, 66.3% choose their menstrual products based on hygiene, and 70.1% rest to alleviate discomfort during their period. These findings for Middle Eastern students were respectively 27.9%; 83.7%; 71.2%; 70.2%; 87.5%; 42.3% and 67.3%, while for African students the figures were 32.3%; 74.2%; 71%; 54.8%; 77.4%; 45.2% and 71% (Table-III).

**Table-III:** Comparison of menstrual experience among university students from Turkic Republics, African, and Middle Eastern Countries.

Menstrual Experiences/Regions		Turkic Republics		Middle Eastern		African		Statistics; p
		n	%	n	%	n	%	
Sensation during First Menstruation	Nothing	203	35.1	29	27.9	10	32.3	No comparison has been made
	Fear	163	28.2	30	28.8	5	16.1	
	Shame	63	10.9	11	10.6	4	12.9	
	Joy	58	10	5	4.8	2	6.5	
	Shame-Fear	46	7.9	25	24.0	8	25.8	
	Joy-Fear	13	2.2	-	-	-	-	
	Shame-Joy	5	0.9	2	1.9	2	6.5	
	Shame-Joy-Fear	-	-	1	1.0	-	-	
	Unspecified	28	4.8	1	1.0	-	-	
	Total	579	100	104	100	31	100	
Receiving Information about Menstrual Period (before it starts)	Yes	491	84.8	87	83.7	23	74.2	2.510;0.285
	No	88	15.2	17	16.3	8	25.8	
	Total	579	100	104	100	31	100	
Getting Information About Perineal Hygiene During Menstruation	Yes	475	82.0	74	71.2	22	71.0	8.161;0.017
	No	104	18.0	30	28.8	9	29.0	
	Total	579	100	104	100	31	100	
Where Did You Learn about Perineal Hygiene During Menstruation*	Mother (Yes)	431	74.4	73	70.2	17	54.8	No comparison has been made
	School (Yes)	164	28.3	9	8.7	7	22.6	
	Internet (Yes)	97	16.8	29	27.9	5	16.1	
	Friend (Yes)	93	16.1	20	19.2	10	32.3	
	Health worker (Yes)	32	5.5	2	1.9	-	-	
	Television (Yes)	28	4.8	9	8.7	4	12.9	
Regular menstruation (in the last year)	Yes	476	82.2	91	87.5	24	77.4	2.381;0.304
	No	103	17.8	13	12.5	7	22.6	
	Total	579	100	104	100	31	100	
Reason for Preferring the Product You Use During Your Menstrual Period*	Hygienic (Yes)	384	66.3	44	42.3	14	45.2	No comparison has been made
	Ease of transportation (Yes)	237	40.9	48	46.2	12	38.7	
	Economic (Yes)	146	25.2	16	15.4	6	19.4	
What do you do to relieve discomfort during your period*	Rest (Yes)	406	70.1	70	67.3	22	71.0	
	Medication use (Yes)	320	55.3	62	59.6	21	67.7	
	Hot shower (Yes)	266	45.9	31	29.8	8	25.8	
	Using herbal products (Yes)	164	28.3	24	23.1	10	32.3	
	Sport (Yes)	57	9.8	-	-	-	-	
	I won’t do anything (Yes)	49	8.5	13	12.5	2	6.5	

n: Number of participants; %: percentile; *χ̅*: Arithmetic mean; SD: Standard deviation; p: significance level. In this table, χ^2^: Chi-square test has been used.

* Multiple items have been marked.

Students from Turkic Republics reported more frequently receiving information about perineal hygiene during menstruation compared to other students, and Middle Eastern students reported this more frequently compared to African students (8.161; 0.017). No significant difference was found in the comparison of receiving information about menstruation before it starts and regular menstruation occurrence among the students participating in the study according to their countries (Table-III).

A comparison of students’ menstrual and genital hygiene practices presents in Table-IV. Differences are observed in genital cleaning in toilets, underwear preferences, pad use during menstruation, sexual activity during menstruation, and other cultural practices. Students from Turkic Republics are found to use cotton underwear more frequently, perform correct genital cleaning in the toilet (front-to-back), and take standing showers during menstruation more often compared to other students (56.086; p:0.000). Students from Turkic Republics and the Middle East are more likely to properly dispose of used menstrual products by wrapping and binning them compared to African students (18.201;0.001). African and Middle Eastern students are more likely to report maintaining sexual activity during menstruation as unspecified compared to students from Turkic Republics (10.116;0.039). It was found that students from Turkic Republics change their menstrual products less frequently than other students; meanwhile, Middle Eastern students change their products more frequently during menstruation compared to African students (Table-IV).

**Table-IV:** Comparison of menstrual and genital hygiene practices among university students from Turkic Republics, African, and Middle Eastern Countries.

Menstrual and Genital Hygiene Practices/Regions		Turkic Republics			Middle Eastern				African					Statistics; p	
		n	%		n	%			n		%				
How to Clean Your Genital Area in the Toilet	From front to back	332	57.3		29	27.9			10		32.3			56.086;0.000	
	From back to front	158	27.3		30	28.8			9		29.0				
	Random	89	15.4		45	43.3			12		38.7				
	Total	579	100		104	100			31		100				
Type of Underwear You Use	Cotton	395	68.2		49	47.1			15		48.4			22.455;0.000 (LH)	
	Nylon	2	0.3		2	1.9			1		3.2				
	Both (Cotton-Nylon)	182	31.4		53	51.0			15		48.4				
	Total	579	100		104	100			31		100				
What Do You Use During Menstruation*	Pad (Yes)	561	96.9		94	90.4			29		93.5			No comparison has been made	
	Cotton (Yes)	27	4.7		22	21.2			5		16.1				
	Tampon (Yes)	23	4.0		1	1.0			-		-				
	Cloth (Yes)	6	1.0		3	2.9			2		6.5				
After Using the Product Used During Menstruation	I wrap it up and throw it in the trash	577	99.7		101	97.1			28		90.3			18.201;0.001(LH)	
	I wash and reuse	1	0.2		2	1.9			-		-				
	I throw it away without wrapping it	1	0.2		1	1.0			3		9.7				
	Total	579	100		104	100			31		100				
Showering During Menstruation	I don’t shower	56	9.7		8	7.7			-		-			68.359;0.000 (LH)	
	Sitting	122	21.1		59	56.7			17		54.8				
	Standing shower	397	68.6		36	34.6			14		45.2				
	Bathtub	3	0.5		-				-		-				
	Unspecified	1	0.2		1	1.0			-		-				
	Total	579	100		104	100			31		100				
Forbidden Practices During Menstruation	Worship	481	83.1		90	86.5			23		74.2			No comparison has been made	
	Showering	18	3.1		4	3.8			1		3.2				
	Worship-Showering	18	3.1		3	2.9			4		12.9				
	Showering-Cooking	7	1.2		6	5.8			1		3.2				
	No newborn visit	2	0.3		-	-			-		-				
	Pickles are untouchable	2	0.3		-	-			-		-				
	No going to the cemetery	2	0.3		-	-			-		-				
	Dough is not kneaded	1	0.2		1	1.0			-		-				
	Animal is not milked	1	0.2		-	-			-		-				
	Unanswered	47	8.1		-	-			2		6.5				
	Total	579	100		104	100			31		100				
Can Sexual Intercourse Continue During Menstruation?	Yes	40	6.9		5	4.8			-		-			10.116;0.039 (LH)	
	No	509	87.9		88	84.6			27		87.1				
	Unspecified	30	5.2		11	10.6			4		12.9				
	Total	579	100		104	100			31		100				
*Frequency of Product Change During Menstrual Period (pcs/day)*		*χ̅*±*S*	*Median*	*1st Quarter; 3rd Quarter*	*χ̅*±*S*	*Median*	*1st Quarter; 3rd Quarter*	*χ̅*±*S*		*Median*		*1st Quarter; 3rd Quarter*		*Statistics;p*	
		4.70±2.02	4.00	3.00; 6.00	6.63±1.84	7.00	6.00; 8.00	6.23±1.52		6.00		5.00; 7.00		81.688;0.000	

n: Number of participants; %: percentile; *χ̅*: Arithmetic mean; SD: Standard deviation; p: significance level; LH: Likelihood Ratio. In this table, χ^2^: Chi-square, Kruskal Wallis-H, and Tamhane tests have been used. *Multiple items have been mark

## DISCUSSION

Menstruation is shaped not only by biological processes but also by socio-cultural norms, which vary across cultures through traditions and taboos.^10^-^12^ Common menstrual symptoms include pain, irritability, appetite changes, and edema.^13^,^14^ These symptoms, prevalent during women’s reproductive years, affect approximately half of all women.^10^,^12^-^14^ Özmermer et al. found moderate to high levels of premenstrual symptoms among university students, with 52.1% reporting PMS and a mean PMS score of 111.36 ± 36.11.^10^

The total MSQ score of students from the Turkic Republics was found to be higher compared to students from the Middle East; also, the average scores for the Menstrual Pain Sub-dimension were higher than those of other students. Seedhom et al. (found a premenstrual symptom prevalence of 80.2% in their study on women in Egypt^15^, while Oo et al. (2016) reported a prevalence of 37.3% in Myanmar^16^, and Vichnin et al. found that 59% of women in New Orleans experienced PMS symptoms.^17^ Sakhi et al. reported that 51.90% of university students in Afghanistan experienced dysmenorrhea.^14^ A study in China observed that cramps were the most common symptom that changed during the acculturation period among international students.^2^ The findings of these studies highlight that symptoms are commonly seen in students before and during menstruation; however, there is variability in the prevalence of menstrual symptoms across different countries. This variability may be due to the different cultural and environmental contexts of the countries where the studies were conducted, as well as differences in the data collection tools and sample sizes used in the research process.

Our study found that students regularly experience menstruation without significant complaints. Karaküçük et al. reported that 51.5% of students experience regular menstruation.^18^ Studies in Nigeria, India, and Afghanistan have reported regular menstruation among women.^11^,^14^,^19^

Menstrual bleeding contains various symbolic meanings that can be conflicting. Studies in Spain and South Cyprus with different generations have shown that younger generations are more knowledgeable about menstruation, more comfortable discussing it, and less likely to adhere to restrictive practices.^20^,^21^ Özarslan Dikmen et al. found in her study in Turkey that young women were less likely to describe menstrual blood as dirty compared to their mothers, saw menstruation not as an illness but as a natural process and were more comfortable discussing menstruation.^22^ This physiological and biological condition of women still manifests as a situation to be ashamed of or hidden in some societies, affecting the presence of women in society.

Female genital mutilation (FGM), involving the cutting or removal of external female genitalia without medical justification, is tied to cultural practices. Among study students, FGM practices were reported by 3.5% from Turkic Republics, 2.9% from the Middle East, and 3.2% from Africa. FGM predominantly affects girls under 14, with high prevalence in countries like Somalia (98%), Guinea (97%), and Djibouti (93%) among women aged 15-49. Regional differences exist; in Senegal, 69.4% of women in the south undergo FGM compared to 6.3% in central regions. Similarly, in Kenya, rates vary from 0.8% in the Western province to 97.5% in the Northeast.^23^-^26^ The research shows that, unfortunately, FGM is still practiced in various countries, albeit with varying frequencies. In addition to these situations, it can be said that the reason why the rate of female circumcision among students from different countries was reported as low in this study is because these students live outside their own country.

The practices during menstruation, the meaning of the menstrual process, socio-economic conditions, or the knowledge acquired during this period can vary from individual to individual. This situation can contribute to a healthier process but also bring about challenges. Our study reveals that students’ experiences related to their menstrual period range from feeling nothing to experiencing fear. Ergeç et al. found that about half of the women described their initial feelings associated with the menstrual cycle as fear, followed by shame and anxiety.^27^ Sapkota et al. found in their study among adolescent girls in Nepal that about one-fifth (19.7%) of the participants expressed a negative attitude towards menstruation as an unwanted condition.^28^

In our study, it was observed that students mostly rest to alleviate discomfort during menstruation. Sakhi et al. found that 31.8% of students believed that taking pain relievers during menstruation harms the menstrual cycle.^14^ Topatan and Kahraman et al. examined the coping methods for premenstrual symptoms in their study and found that 57% of students experienced mood changes, and 27.2% reported being unable to cope with these symptoms.^29^ Other participants engaged in activities that made them happy, such as reading books, watching TV, listening to music (20.1%), sleeping (22%), crying, praying, thinking positive thoughts, or chatting (17.4%).^29^

Students from Turkic Republics change their menstrual products less frequently compared to other students; students from the Middle East change them more frequently than those from Africa. Gün and Adana et al. found in their study with working adolescents that 68.8% of participants changed their pads or clothes whenever they felt it was wet.^30^ A study in South India reported that 78.3% of girls changed their pads 2-3 times per day during their menstrual period.^31^

Our study found that most female students prefer to use disposable pads. Similarly, studies in the literature report that the use of pads or sanitary napkins is prevalent during menstruation.^14^,^18^,^32^,^33^ The disposal of used products in both this study and the literature generally involves throwing them in the trash.^5^,^33^

In this research, it was reported that students generally prefer cotton underwear, the majority shower during menstruation, and students from Turkic Republics perform genital cleaning from front to back, while students from the Middle East and Africa perform it haphazardly. Hygienic practices are important for genital health. Studies with university students show that bathing during menstruation is a common habit,^14^,^33^,^34^ and they often prefer cotton underwear.^32^,^35^ Türkmen and Karagüzel et al. reported that genital cleaning is more frequently done from front to back.^34^

In our study, most students learned about menstruation from their mothers. Similarly, Sapkota et al. (2013) found mothers (39.3%) were the primary source, followed by sisters and friends (18%).^28^ Ergeç et al.^27^ reported mothers informed 76.5% of women, with siblings, friends, and schools also contributing.^27^ Sources of information are crucial for adolescent girls because accurate information plays a vital role in how they experience and manage their first menstruation and subsequent cycles.

### Strength of the Study:

This study highlights cross-cultural differences in menstrual symptoms and practices among female university students in Türkiye, particularly from low-income countries, addressing a gap in the literature. Its strength lies in its diverse sample. In addition, the study reveals the effects of cultural characteristics on menstrual health and provides important information for the creation of culturally adapted health services and education plans.

### Limitations

However, limitations include limited generalizability due to from a single university sampling, reliance on self-reported data, and the educational level of participants. Non-responses to some items and the need for broader studies involving women with lower socioeconomic and educational backgrounds are noted.

## CONCLUSION

The research has shown that there are international differences in the symptoms and practices related to menstruation among female students from different cultures and nationalities. It was found that students from Turkic Republics, the Middle East, and Africa have similar and above-average menstrual symptoms; female genital mutilation is still practiced in countries, albeit with varying frequency; the frequency of changing menstrual products differs between regions; in some countries, the onset of menstruation in girls is considered a condition that should be kept secret; girls are afraid when menstruation starts; information is primarily obtained from mothers before menstruation begins; pads are mostly used during menstruation; cotton underwear is preferred during this period; bathing is possible during menstruation; and practicing religious rituals is prohibited during menstruation.

### Recommendations

Given that menstruation holds a significant place in the lives of all women, universal efforts, especially led by health professionals, should be developed and sustained to instill correct, reliable, and healthy behaviors in girls, particularly those entering adolescence, from the early years of adolescence to ensure a healthy menstruation period. Additionally, providing information about reproductive health services in university counseling and guidance centers helps students make informed decisions about their reproductive health, reduces anxiety, and increases their self-confidence. This supports both their physical and psychological well-being.

### Authors Contributions:

**DY, SK, FA:** Idea/Concept.

**DY, SK, FA, AM:** Design.

**SK:** Data Collection.

**DY, SK, FA, AM:** Audit/Consultancy.

**DY, SK, FA:** Analysis and/or Interpretation.

**DY, SK, FA, SÖ, CVA, AM:** Source Literature, Writing the Article.

**FA, AM:** Critical Review

All authors, especially the corresponding author, are responsible for the accuracy and integrity of this manuscript. All authors have read the final version of the manuscript and approved it.
